# Outcomes of Technically Optimal Intraoperative Radiation Therapy With Electrons Versus Whole-Breast External Beam Radiation in Suitable Patients With Breast Cancer

**DOI:** 10.1016/j.adro.2026.102086

**Published:** 2026-05-30

**Authors:** Tanun Jitwatcharakomol, Jiraporn Setakornnukul, Suebwong Chuthapisith, Adune Ratanawichitrasin, Janjira Petsuksiri, Kullathorn Thephamongkhol

**Affiliations:** a Division of Radiation Oncology, Department of Radiology, Faculty of Medicine Siriraj Hospital, Mahidol University, Bangkok, Thailand; b Division of Head-Neck and Breast Surgery, Department of Surgery, Faculty of Medicine Siriraj Hospital, Mahidol University, Bangkok, Thailand

## Abstract

**Purpose:**

Intraoperative radiation therapy (IORT) with electrons is associated with higher rates of ipsilateral breast tumor recurrence in early-stage breast cancer than whole-breast external beam radiation therapy (EBRT). Although limited randomized and retrospective data suggest that this approach may suit a subset of patients, its role remains uncertain. This study aimed to compare oncological outcomes between technically optimal IORT with electrons and whole-breast EBRT in suitable patients with breast cancer.

**Methods and Materials:**

In this single-center retrospective cohort study, patients were grouped by radiation treatment: IORT with electrons or whole-breast EBRT. Endpoints included disease-free survival (DFS), local control, and breast cancer–specific survival (BCSS).

**Results:**

From January 2011 to December 2021, 204 eligible patients were reviewed (IORT, 74; EBRT, 130). The median follow-up was 5.1 years. The 5-year DFS rates were 95.61% for IORT and 98.08% for EBRT (log-rank *P* = .02). The 5-year local control rates were 98.46% for IORT and 100% (no event) for EBRT (log-rank *P* = .07). The 5-year BCSS rates were comparable: 98.61% for IORT and 100% (no event) for EBRT (log-rank *P* = .23). On univariable analysis for DFS, receipt of systemic treatment was a statistically significant protective factor (hazard ratio, 0.09; 95% CI, 0.01-0.74). Receipt of whole-breast EBRT was associated with a reduced risk of events, although this did not reach statistical significance (hazard ratio, 0.12; 95% CI, 0.02-1.01).

**Conclusions:**

IORT with electrons was associated with a modest but statistically significant reduction in 5-year DFS in appropriately selected patients compared with whole-breast EBRT, whereas BCSS remained comparable between groups. Therefore, electron-based IORT should be approached with caution and reserved for patients who prioritize treatment convenience.

## Introduction

Breast cancer is the most common cancer among women worldwide, and its incidence increases each year. Because adjuvant radiation therapy can substantially reduce recurrence and mortality,^1^ patients with breast cancer often constitute a considerable proportion of radiation therapy department caseloads. To alleviate this burden, researchers have developed new breast radiation therapy regimens. Drawing on principles of tumor radiobiology,^2^ hypofractionated regimens were proposed and tested in multiple large-scale randomized controlled trials.^3^, ^4^, ^5^, ^6^, ^7^, ^8^, ^9^ Partial breast irradiation has been proposed to further reduce treatment volume and duration in selected patients with early-stage breast cancer. Intraoperative radiation therapy (IORT) represents an extreme form of partial breast irradiation, delivering a single radiation fraction directly to the tumor bed during breast-conserving surgery. This approach offers unparalleled convenience while minimizing irradiation of the surrounding normal breast tissue.

The ELIOT (Electron Intraoperative Therapy) trial^10^ randomized patients with early-stage breast cancer undergoing breast-conserving surgery to adjuvant IORT with 21 Gy electrons or external beam radiation therapy (EBRT). The IORT group had a higher 5-year ipsilateral breast tumor recurrence (IBTR) than the EBRT group (4.4% vs 0.4%; hazard ratio [HR], 9.3; 95% CI, 3.3-26.3). However, a subsequent retrospective review by Leonardi et al^11^ stratified ELIOT trial patients according to the American Society for Radiation Oncology (ASTRO)^12^ definition as suitable, cautionary, or unsuitable. The 5-year recurrence rate among suitable patients was only 1.5%, supporting the use of IORT in this select patient population.^12^^,^^13^

The controversy resurfaced with the 2021 update of the ELIOT trial.^14^ The IBTR rate difference between IORT and EBRT widened considerably, with 15-year rates of 12.6% for IORT and 2.4% for EBRT. Notably, even the suitable subgroup per the ASTRO definition demonstrated high recurrence, with 10-year and 15-year IBTR rates of 6.1% and 13.1%, respectively. These findings raised questions about whether inferior local control with IORT reflects patient selection limitations, technical shortcomings in delivery, or an inherent limitation of partial versus whole-breast irradiation.

Despite efforts to optimize patient selection, local recurrence rates remain considerably higher with IORT than with EBRT. To address this, investigators have examined the technical aspects of IORT delivery. A Belgian study^15^ reported a 5-year local recurrence rate of 2.7% among patients with early-stage breast cancer treated with IORT using an average electron cone size of 5.5 cm for tumors ≤2 cm. This rate is lower than the 4.2% reported in the ELIOT trial, suggesting that suboptimal technique rather than partial breast irradiation itself may account for the inferior outcomes in prior IORT studies.

Therefore, our study combines careful patient selection with technically optimized electron-based IORT, aiming to compare oncological outcomes between IORT and whole-breast EBRT in suitable patients with breast cancer per the ASTRO definition.^12^

## Methods and Materials

### Study population

Eligible patients had newly diagnosed, pathologically confirmed early-stage breast cancer from January 2011 to December 2021, underwent breast-conserving surgery with axillary lymph node biopsy or dissection, and met all ASTRO-suitable criteria^12^ (Table 1). Exclusion criteria included distant metastasis (per the American Joint Committee on Cancer Staging Manual, 8th edition), bilateral breast cancer, multiple primary cancers, and prior chest wall or axillary radiation.

**Table 1:** American Society for Radiation Oncology (ASTRO) suitability criteria for partial breast irradiation patient selection

Criterion	Requirement
Age	≥50 y
Tumor size	≤2 cm
Histology	Invasive ductal, mucinous, tubular, papillary, medullary, or colloid carcinoma
Grade	Any
Focality	Clinically unifocal
Centricity	Unicentric
Margins	≥2 mm
LVSI	None
EIC	None
ER status	Positive
Nodal status	pN0
Neoadjuvant therapy	None
Genetics	*BRCA1/2* negative
DCIS	≤2.5 cm, screen-detected, low-to-intermediate grade, margin ≥3 mm

*Abbreviations: BRCA1/2* = breast cancer gene 1/2; DCIS = ductal carcinoma in situ; EIC = extensive intraductal component; ER = estrogen receptor; LVSI = lymphovascular space invasion; pN0 = pathologic node-negative.

Pure DCIS has been permitted since 2016.

**Figure 1:**
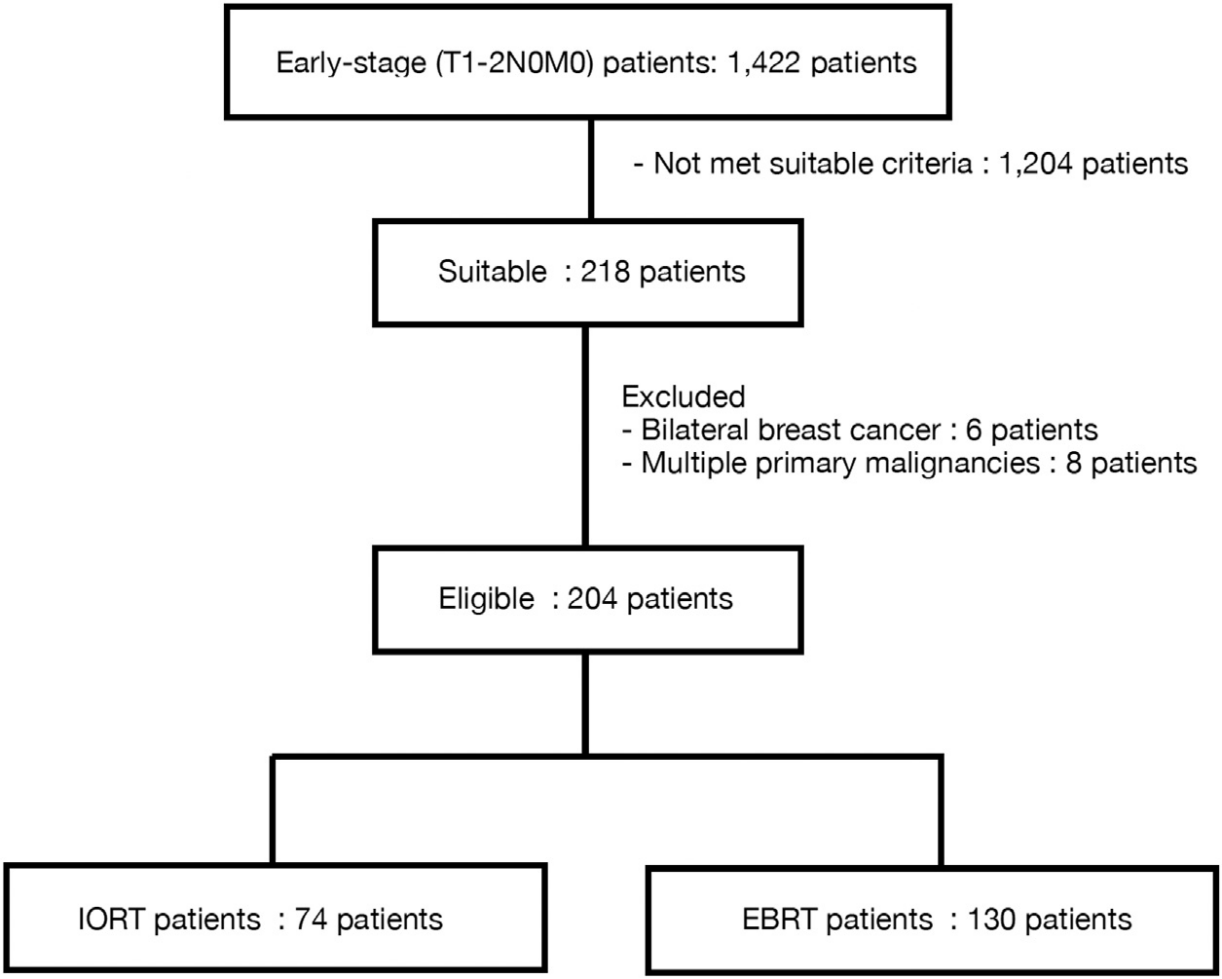
CONSORT flow diagram of patient enrollment and group allocation. Patients with early-stage (T1-2N0M0) breast cancer were screened against the American Society for Radiation Oncology suitability criteria. Eligible patients were allocated to intraoperative radiation therapy (IORT) or external beam radiation therapy (EBRT). *Abbreviation*: CONSORT = Consolidated Standards of Reporting Trials.

All patients with breast cancer diagnosed from January 2011 to December 2021 were reviewed. Of 1422 patients, 204 were included (IORT, 74; EBRT, 130) (Fig. 1).

After treatment, patients were seen at 3- to 4-month intervals for the first 2 years and at 6-month intervals thereafter, with annual mammography. Additional imaging was performed as clinically indicated.

### Study design

This single-center retrospective study was conducted at Siriraj Hospital, Thailand and approved by the Siriraj Ethical Review Board. Patients were divided into 2 groups based on radiation technique: IORT with electrons or whole-breast EBRT.

In the IORT group, a Mobetron device (Intraop Medical Inc) was used to deliver 21 Gy electrons to the 90% isodose line. The treatment field encompassed the tumor bed with an additional 1.5 to 2.0 cm margin in each dimension. Sentinel lymph node biopsy with frozen-section analysis was performed for each patient to confirm negative nodal status before IORT.

In the whole-breast EBRT group, all patients received linear accelerator-based radiation therapy. Prescribed doses were 50 Gy in 25 fractions, 42.5 Gy in 16 fractions, or 40 Gy in 15 fractions. Hypofractionated regimens (42.5 Gy in 16 fractions or 40 Gy in 15 fractions) were increasingly adopted in later years, after long-term evidence^3^^,^^5^ demonstrated comparable efficacy and safety to conventional fractionation. A tumor bed boost was permitted at the physician’s discretion, with doses of 8 to 14 Gy in 3 to 7 fractions.

### Statistical analysis

The outcomes were disease-free survival (DFS), local control, and breast cancer–specific survival (BCSS), all measured from the start of radiation therapy. DFS was defined as the time to any recurrence or death from any cause, local control as the time to IBTR, and BCSS as the time to breast cancer–related death.

Baseline clinicopathological characteristics were compared between the 2 treatment groups using the Fisher exact test for categorical variables and the *t* test or the Mann-Whitney *U* test for continuous variables, as appropriate. DFS, local control, and BCSS were estimated using the Kaplan-Meier method and compared between groups using the log-rank test; patients without events were censored at the date of last follow-up. Univariable Cox proportional hazards regression was performed to identify factors associated with outcomes; multivariable analysis was not performed because of the low number of events. All analyses were conducted using Stata release 18 (StataCorp LLC).

## Results

### Baseline characteristics

Patient and treatment characteristics are summarized in Table 2, Table 3. The mean tumor size was 1.1 cm in the IORT group and 1.22 cm in the EBRT group (*P* = .06). Most patients in both groups had invasive ductal carcinoma (92.6%) with grade 2 tumors (59.3%), and all were estrogen receptor–positive. Progesterone receptor–positive was 94.6% in the IORT group and 89.2% in the EBRT group (*P* = .30). Most patients in both groups received systemic hormonal therapy (98%), whereas chemotherapy was administered in 26.6% of the EBRT group compared with 11.1% of the IORT group (*P* = .08). Among those who received chemotherapy (8 patients in the IORT group and 34 patients in the EBRT group), human epidermal growth factor receptor 2–positive status was observed in 2 patients (25%) and 9 patients (26.5%), respectively.

**Table 2:** Baseline patient and tumor characteristics by treatment group

Characteristic	Total (N = 204)	IORT (n = 74)	EBRT (n = 130)	*P* value
Age, y, mean (SD)	61.30 (7.34)	63.83 (6.52)	59.85 (7.41)	<.01
Breast laterality				
Right	108 (52.9)	41 (55.4)	67 (51.5)	.66
Left	96 (47.1)	33 (44.6)	63 (48.5)	
Tumor size, cm, mean (SD)	1.17 (0.46)	1.10 (0.47)	1.22 (0.46)	.06
Tumor location				
Upper inner	57 (27.9)	21 (28.4)	36 (27.7)	.85
Upper outer	97 (47.5)	33 (44.6)	64 (49.2)	
Lower inner	13 (6.4)	4 (5.4)	9 (6.9)	
Lower outer	24 (11.8)	11 (14.9)	13 (10.0)	
Central	13 (6.4)	5 (6.8)	8 (6.2)	
Histologic grade				
G1	66 (32.4)	26 (35.1)	40 (30.8)	.81
G2	121 (59.3)	42 (56.8)	79 (60.8)	
G3	17 (8.3)	6 (8.1)	11 (8.5)	
Histology				
IDC	189 (92.6)	72 (97.3)	117 (90.0)	.04
Other	13 (6.4)	1 (1.4)	12 (9.2)	
Pure DCIS	2 (1.0)	1 (1.4)	1 (0.8)	
Tumor focality				
Single	202 (99.0)	73 (98.6)	129 (99.2)	1.00
Multiple	2 (1.0)	1 (1.4)	1 (0.8)	
ER status	204 (100.0)	74 (100.0)	130 (100.0)	N/A
PR status				
Negative	18 (8.8)	4 (5.4)	14 (10.8)	.30
Positive	186 (91.2)	70 (94.6)	116 (89.2)	
HER2 status				
Negative	181 (88.7)	66 (89.2)	115 (88.5)	.91
Equivocal	7 (3.4)	3 (4.1)	4 (3.1)	
Positive	13 (6.4)	4 (5.4)	9 (6.9)	
N/A	3 (1.5)	1 (1.4)	2 (1.5)	
Ki-67, %, mean (SD)	22.03 (15.71)	19.37 (12.08)	23.37 (17.15)	.12
Intrinsic subtype				
Luminal A	54 (26.5)	18 (24.3)	36 (27.7)	.21
Luminal B	111 (54.4)	37 (50.0)	74 (56.9)	
Luminal (unclassified A/B)	39 (19.1)	19 (25.7)	20 (15.4)	
DCIS component				
No	86 (42.2)	30 (40.5)	56 (43.1)	.77
Yes	118 (57.8)	44 (59.5)	74 (56.9)	
DCIS grade				
G1	12 (10.2)	5 (11)	7 (9)	.91
G2	80 (67.8)	30 (68)	50 (68)	
N/A	26 (22.0)	9 (20)	17 (23)	
Systemic treatment				
No	2 (1.0)	2 (2.7)	0 (0.0)	.12
Yes	200 (98.0)	72 (97.3)	128 (98.5)	
N/A	2 (1.0)	0 (0.0)	2 (1.5)	
Systemic regimen				
HT alone	158 (79.0)	64 (88.9)	94 (73.4)	.08
CMT alone	3 (1.5)	0 (0.0)	3 (2.3)	
CMT + HT	37 (18.5)	8 (11.1)	29 (22.7)	
CMT + HT + trastuzumab	1 (0.5)	0 (0.0)	1 (0.8)	
CMT + trastuzumab	1 (0.5)	0 (0.0)	1 (0.8)	
ASTRO (2023) category				
Recommended	180 (88.2)	66 (89.2)	114 (87.7)	.94
Conditionally recommended	13 (6.4)	4 (5.4)	9 (6.9)	
Conditionally not recommended	11 (5.4)	4 (5.4)	7 (5.4)	

*Abbreviations:* ASTRO = American Society for Radiation Oncology; CMT = chemotherapy; DCIS = ductal carcinoma in situ; EBRT = external beam radiation therapy; ER = estrogen receptor; G = grade; HER2 = human epidermal growth factor receptor 2; HT = hormonal therapy; Ki-67 = marker of proliferation Ki-67; IDC = invasive ductal carcinoma; IORT = intraoperative radiation therapy; N/A = not applicable; PR = progesterone receptor.

Data are presented as n (%) unless otherwise indicated. *P* values were calculated using the Fisher exact test for categorical variables and the *t* test or Mann-Whitney *U* test for continuous variables, as appropriate.

**Table 3:** Radiation treatment parameters by treatment group

Parameter	Value
**IORT group (n = 74)**	
Tumor depth, cm, mean (SD)	2.86 (0.80)
Chest wall depth, cm, mean (SD)	3.86 (0.92)
Tumor bed isodose, %, mean (SD)	93.35 (7.19)
Chest wall isodose, %, mean (SD)	65.7 (21.8)
Bolus use	
No	59 (80)
Yes	15 (20)
Shielding use	
No	4 (5)
Yes	70 (95)
Electron energy (MeV)	
6	14 (19)
9	17 (23)
12	43 (58)
Cone size (cm)	
5	6 (8)
5.5	11 (15)
6	30 (41)
6.5	4 (5)
7	23 (31)
EBRT group (*n* = 130)	
Radiation technique	
2D	26 (20)
3D conformal	103 (79.2)
IMRT/VMAT	1 (0.8)
DIBH	
No	100 (96.2)
Yes	4 (3.8)
Prescribed dose	
50 Gy/25 Fx	63 (48.5)
42.5 Gy/16 Fx	64 (49.2)
40 Gy/15 Fx	3 (2.3)
Sequential tumor bed boost	
No	81 (62.3)
Yes	49 (37.7)
Boost dose (Gy)	
8	13 (26)
10	35 (72)
14	1 (2)
Mean heart dose,* Gy, mean (SD)	1.93 (1.63)
Mean total lung dose,* Gy, mean (SD)	4.38 (2.20)

*Abbreviations:* 2D = 2-dimensional; 3D = 3-dimensional; DIBH = deep inspiratory breath hold; EBRT = external beam radiation therapy; Fx = fractions; IMRT = intensity modulated radiation therapy; IORT = intraoperative radiation therapy;VMAT = volumetric modulated arc therapy.

⁎ Heart and lung dose data are from patients treated with 3D conformal or IMRT/VMAT only. Data are presented as n (%) unless otherwise indicated.

### Radiation treatment details

In the IORT group, the mean tumor depth was 2.86 cm, the mean chest wall depth was 3.86 cm, and tumor beds received a mean isodose of 93.35%. Most patients did not use a bolus (80%) but did use shielding (95%). The most common energy and cone size were 12 MeV and 6 cm, respectively. In the EBRT group, the most common technique was 3-dimensional radiation therapy without deep inspiratory breath hold (79.2%). The most commonly prescribed regimens were 50 Gy/25 fractions (Fx) and 42.5 Gy/16 Fx (97.7%), with a sequential boost in 37.7% of patients. The mean heart and lung doses were 1.93 Gy and 4.38 Gy, respectively.

### Survival and recurrence outcomes

**Figure 2:**
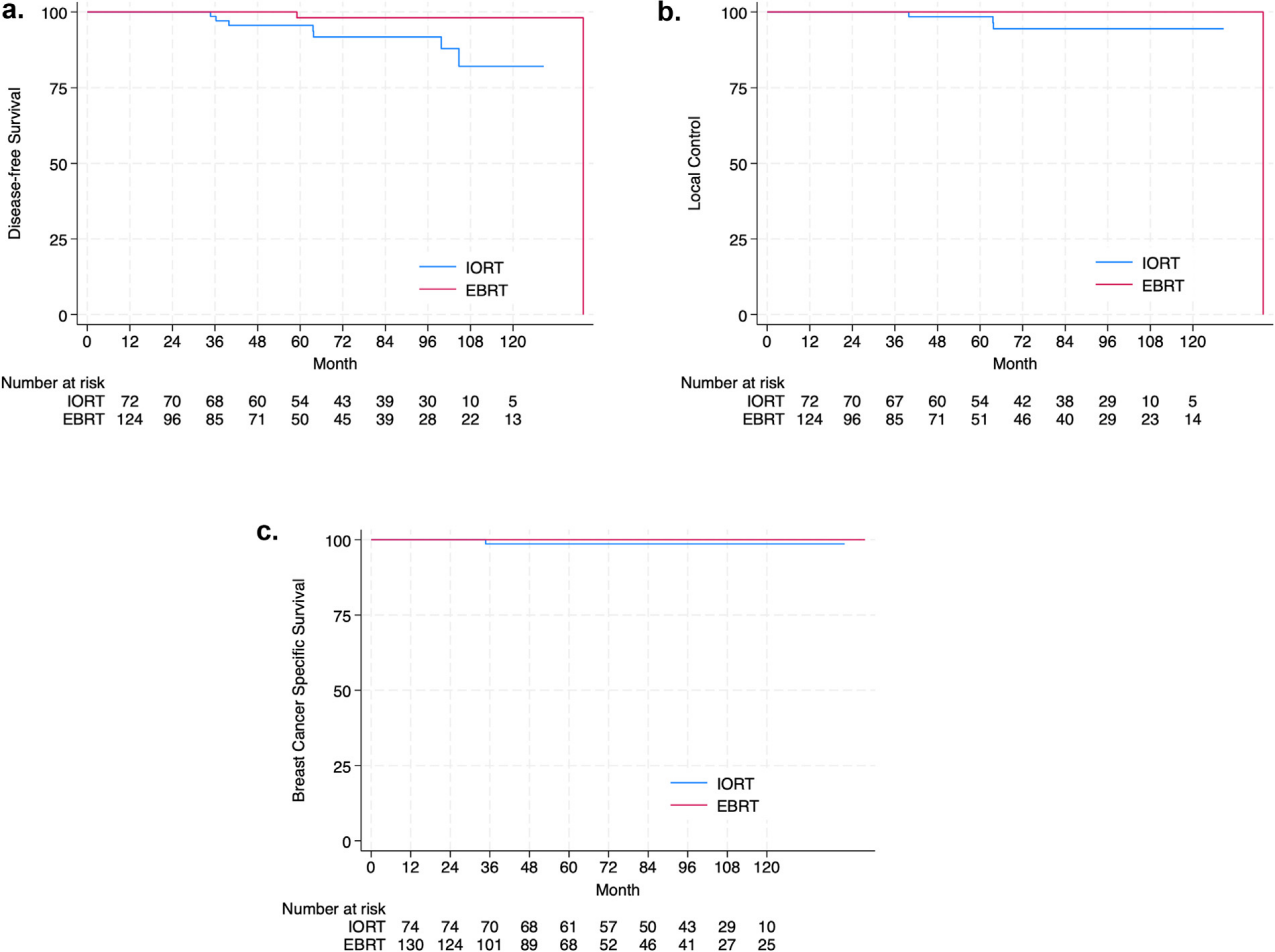
(a) Kaplan-Meier curve for disease-free survival by treatment group (log-rank *P* = .02). The number at risk at each time point is shown below the x-axis. (b) Kaplan-Meier curve for local control by treatment group (log-rank *P* = .07). The number at risk at each time point is shown below the x-axis. (c) Kaplan-Meier curve for breast cancer–specific survival by treatment group (log-rank *P* = .23). The number at risk at each time point is shown below the x-axis. *Abbreviations:* EBRT = external beam radiation therapy; IORT = intraoperative radiation therapy.

The median follow-up was 5.1 years overall, 7.6 years for the IORT group, and 4.3 years for the EBRT group. The 5-year DFS rates were 95.61% for IORT and 98.08% for EBRT (log-rank *P* = .02), and the 5-year local control rates were 98.46% for IORT and 100% (no event) for EBRT (log-rank *P* = .07). The corresponding 5-year absolute differences between the EBRT and IORT groups were 2.47% for DFS and 1.54% for local control. The 5-year BCSS rates were comparable: 98.61% for IORT and 100% (no event) for EBRT (log-rank *P* = .23; Fig. 2).

### Outcomes by updated ASTRO classification

According to the updated ASTRO Clinical Practice Guideline on partial breast irradiation (2023), 180 patients (88.2%) in the overall cohort were categorized as “recommended for partial breast irradiation,” 13 (6.4%) as “conditionally recommended,” and 11 (5.4%) as “conditionally not recommended” (Table 2). The primary reason for classification into the conditionally not recommended group was human epidermal growth factor receptor 2–positive status without receipt of trastuzumab. After excluding this group, the 5-year DFS rate was statistically significantly higher for EBRT (97.87%) than for IORT (96.87%; log-rank *P* = .04). However, the 5-year local control rates showed no statistically significant difference between IORT (98.39%) and EBRT (100%, no event; log-rank *P* = .08; Figs. E1 and E2).

### Patterns of first failure

Patterns of first failure are detailed in Tables E1 and E2. Local failure occurred in 3 patients (4%) in the IORT group and 1 patient (0.8%) in the EBRT group, all within the index quadrant. In addition, 2 patients (2.7%) in the IORT group and 1 patient (0.8%) in the EBRT group experienced regional failure; no patients experienced distant recurrence.

### Univariable analysis

Univariable analysis results for DFS are presented in Table 4. Receipt of systemic treatment was a statistically significant protective factor (HR, 0.09; 95% CI, 0.01-0.74). Receipt of whole-breast EBRT was associated with a reduced risk of events, although this did not reach statistical significance (HR, 0.12; 95% CI, 0.02-1.01). Multivariable analysis was not performed because of the low number of events.

**Table 4:** Univariable Cox proportional hazards regression analysis for disease-free survival

Variable	HR (95% CI)	*P* value
Radiation technique		
IORT	Ref	
EBRT	0.12 (0.02-1.01)	.05
Tumor size (cm)	2.65 (0.55-12.65)	.22
Histologic grade		
G1	Ref.	
G2/3	1.97 (0.39-9.80)	.41
Intrinsic subtype		
Luminal A	Ref.	
Nonluminal A	2.98 (0.36-24.26)	.31
Systemic treatment		
No	Ref.	
Yes	0.09 (0.01-0.74)	.03*

*Abbreviations:* EBRT = external beam radiation therapy; G = grade; HR = hazard ratio; IORT = intraoperative radiation therapy; Ref. = reference category.*Statistically significant at *P* < .05.

Multivariable analysis was not performed because of the low number of events.

## Discussion

With a median follow-up of 5.1 years, 5-year DFS was statistically significantly lower in the IORT group than in the whole-breast EBRT group, with an absolute difference of 2.47%. Our IORT protocol used the electron technique described in the ELIOT trial but incorporated stricter patient selection and careful choice of cone size to cover the tumor bed with an additional 1.5 to 2.0 cm margin in each dimension. Despite these refinements, DFS remained lower in the IORT group than in the whole-breast EBRT group.

These results are consistent with the updated ELIOT trial,^14^ which reported higher IBTR in the IORT group than in the whole-breast EBRT group: 4.2% versus 0.5% at 5 years and 12.6% versus 2.4% at 15 years. Subgroup analysis of ASTRO-suitable patients yielded similar findings, with 5-year and 15-year IBTR rates of 2% and 13.1%, respectively, substantially higher than in the whole-breast EBRT group.

Because of its retrospective design, this study is subject to selection bias between the 2 groups. The EBRT group appeared to have more adverse features associated with local relapse and distant metastasis, such as younger age and larger tumor size. Despite these unfavorable characteristics in the EBRT group, DFS remained lower in the IORT group. We therefore examined the characteristics of treatment failures (Table E2) and found no inappropriate treatment or high-risk features to explain the inferior outcomes. These findings further underscore concerns about the technical limitations of IORT with electrons, as we have previously discussed.^16^

Although local failures may have occurred by chance, the incidence was disproportionately higher in the IORT group than in the whole-breast EBRT group (4% vs 0.8%). The likelihood of true local recurrence is further supported by the finding that all recurrent tumors arose in the same quadrant as the index tumor. Unlike EBRT, IORT does not generate a dose-volume histogram or provide visual confirmation of dose distribution, limiting the ability to verify target coverage. Consequently, geographic miss or target underdosage may occur during treatment.

An additional challenge is that IORT delivers treatment in a single fraction, meaning that some tumor cells may be in less radiosensitive phases of the cell cycle at the time of irradiation.^17^ This could translate into inferior outcomes compared with fractionated whole-breast EBRT.

Given the higher failure rate with the IORT technique, the use of electron-based IORT at our institution has declined over time (Fig. E3). As a result, the EBRT group had a shorter median follow-up of 4.3 years compared with 7.6 years in the IORT group, a disparity that may partially account for the higher incidence of adverse oncological outcomes in the IORT cohort. Notably, 2 of 3 patients treated with IORT who experienced local failure did so beyond 5 years of follow-up. Another important confounder is systemic treatment, which was statistically significant on univariable analysis. Patients in the EBRT group received more chemotherapy, which could substantially reduce locoregional and distant failures, resulting in improved DFS.

Lastly, the LUMINA study demonstrated that omitting adjuvant radiation therapy may be reasonable for a highly selected, low-risk population with luminal A breast cancer. In that trial, patients aged ≥55 years with T1N0, grade 1 to 2 tumors, and a Ki-67 index <13.25% had a 5-year local recurrence rate of only 2.3% (90% CI, 1.3%-3.8%).^18^ In very favorable patients, therefore, omission of radiation therapy may be an appropriate alternative to IORT, preserving the option of whole-breast EBRT for local recurrence while maintaining breast conservation. Nevertheless, IORT may still offer a meaningful option for patients with limited access to EBRT who desire adjuvant radiation therapy. Patient preferences, tumor biology, and access to care vary considerably among candidates for IORT, and careful patient selection with preoperative multidisciplinary consultation involving surgical and radiation oncology is essential to support informed, individualized decision-making.

A key limitation of our study is the relatively short follow-up, which is particularly important in this clinical context because, as demonstrated in the ELIOT trial, differences in outcomes may become more pronounced over time. The retrospective design inherently carries the risk of selection bias and confounding factors that may affect both the results and their interpretation. Although recent publications have emphasized the importance of optimal IORT technique,^15^ these reports were published after the treatment period of the present cohort. Nevertheless, all IORT procedures in our study were performed according to institutional protocols that reflected standard clinical practice at the time. No obvious technical deficiencies were identified on retrospective review; however, unrecognized technical variability cannot be completely excluded.

Given the inferior 5-year DFS in the IORT group compared with the whole-breast EBRT group—even among patients deemed suitable and treated with an appropriate cone size—IORT with electrons should be applied with caution. Shared decision-making is essential when considering electron-based IORT.

## Conclusion

IORT with electrons was associated with a modest but statistically significant reduction in 5-year DFS in appropriately selected patients compared with whole-breast EBRT, whereas BCSS remained comparable between groups. Therefore, electron-based IORT should be approached with caution and reserved for patients who prioritize treatment convenience.

## Disclosures

The authors declare that they have no known competing financial interests or personal relationships that could have appeared to influence the work reported in this paper.

## Appendix Supplementary materials

Supplementary_V3 Image, application 1

## Footnotes

Supplementary material associated with this article can be found in the online version at doi:10.1016/j.adro.2026.102086.
