# Supplementary material for: Outcomes of Technically Optimal Intraoperative Radiation Therapy With Electrons Versus Whole-Breast External Beam Radiation in Suitable Patients With Breast Cancer
Source: Adv Radiat Oncol. 2026 May 30;11(11):102086. doi: 10.1016/j.adro.2026.102086 (PMC13380423; doi:10.1016/j.adro.2026.102086)
Supplement: Supplementary_V3 [file mmc1.pdf]

Supplementary Tables and Figures

Supplementary Table 1 Pattern of first failure by treatment group

Supplementary Table 2 Clinical and treatment characteristics of patients with disease recurrence

Supplementary Figure 1 Kaplan–Meier curve for disease-free survival among recommended and conditionally recommended patients.

Supplementary Figure 2 Kaplan–Meier curve for local control among recommended and conditionally recommended patients.

Supplementary Figure 3 Total eligible patients per year by radiation technique ($N = 204$).

Supplementary Table 1 Pattern of first failure by treatment group

Pattern of first failure	IORT (*n* = 74)	EBRT (*n* = 130)
Local	3 (4.0%)	1 (0.8%)
Regional	2 (2.7%)	1 (0.8%)
Distant	0 (0.0%)	0 (0.0%)
Total	**5 (6.7%)**	**2 (1.5%)**

Data are presented as *n* (%). First failure is defined as the first documented recurrence event after completion of radiation therapy. No distant recurrences were observed in either group.

Abbreviations: EBRT, external beam radiation therapy; IORT, intraoperative radiation therapy

Supplementary Table 2 Clinical and treatment characteristics of patients with disease recurrence

Patient No.	Treatment group	Initial tumor quadrant	Recurrence location	Time to failure, mo	Tumor size, cm	Margin, mm	Grade	ER	PR	Systemic treatment	Systemic regimen	IORT cone, cm	IORT energy, Mev	EBRT technique	EBRT dose	EBRT field	Boost
Local recurrence																	
1	EBRT	UIQ	UIQ	139.8	0.6	10	G1	+	+	Yes	HT alone	–	–	2D	50Gy/25F	Breast	No
2	IORT	UOQ	UOQ	63.8	1.5	5	G2	+	–	Yes	HT alone	5.5	12	–	–	–	–
3	IORT	UIQ	UIQ	39.9	1.7	4	G2	+	+	Yes	HT alone	6.0	9	–	–	–	–
4	IORT	UIQ	UIQ	63.6	1	7	G1	+	+	Yes	HT alone	6.0	12	–	–	–	–
Regional recurrence																	
1	EBRT	UIQ	Axillary LN	59.1	1.1	4	G2	+	+	Yes	CMT + HT	–	–	2D	50Gy/25F	Breast	Yes
2	IORT	LOQ	Axillary LN	36.3	2	3	G1	+	+	Yes	HT alone	6.0	6	–	–	–	–
3	IORT	UIQ	Axillary LN	26.6	1.3	2	G2	+	+	No	–	6.0	9	–	–	–	–

Each row represents 1 patient with a documented recurrence event. All local recurrences occurred in the same quadrant as the index tumor. The en dash (–) indicates not applicable (parameter pertains to the other treatment group). The plus sign (+) indicates positive; the en dash (–) under ER/PR indicates negative

Abbreviations: 2D, 2-dimensional; CMT, chemotherapy; EBRT, external beam radiation therapy; ER, estrogen receptor; Fx, fractions; G, grade; HT, hormonal therapy; IORT, intraoperative radiation therapy; LN, lymph node; LOQ, lower outer quadrant; MeV, mega-electron volts; mo, months; PR, progesterone receptor; UIQ, upper inner quadrant; UOQ, upper outer quadrant

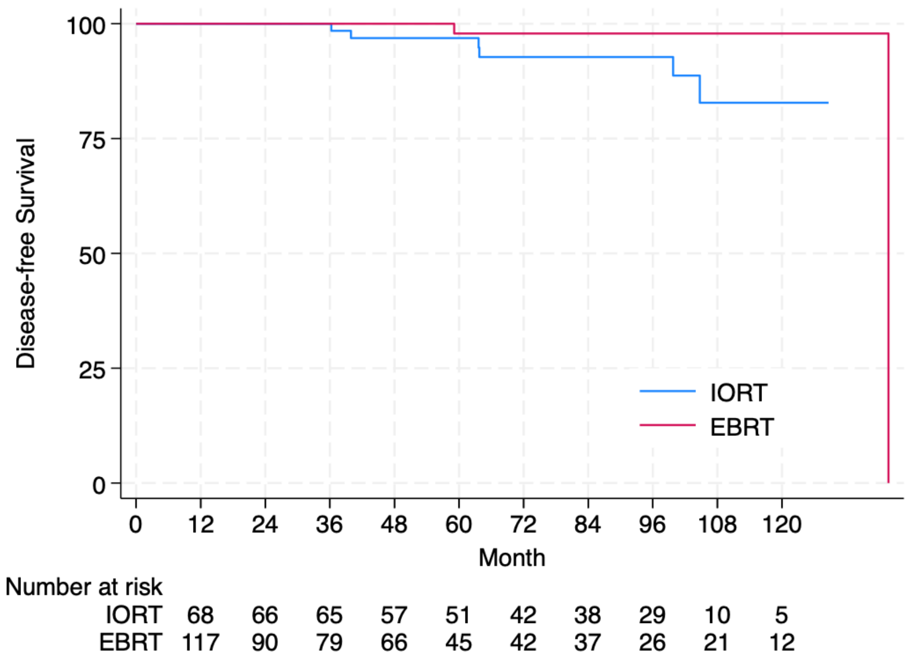

Supplementary Figure 1 Kaplan–Meier curve for disease-free survival among recommended and conditionally recommended patients per the 2023 ASTRO guideline on partial breast irradiation ($N = 193$; log-rank $P = 0.04$). Patients classified as conditionally not recommended per the 2023 American Society for Radiation Oncology guideline were excluded. The 5-year disease-free survival rates were 96.87% for the IORT group and 97.87% for the EBRT group. The number at risk at each time point is shown below the x-axis.

Abbreviations: ASTRO, American Society for Radiation Oncology; DFS, disease-free survival; EBRT, external beam radiation therapy; IORT, intraoperative radiation therapy

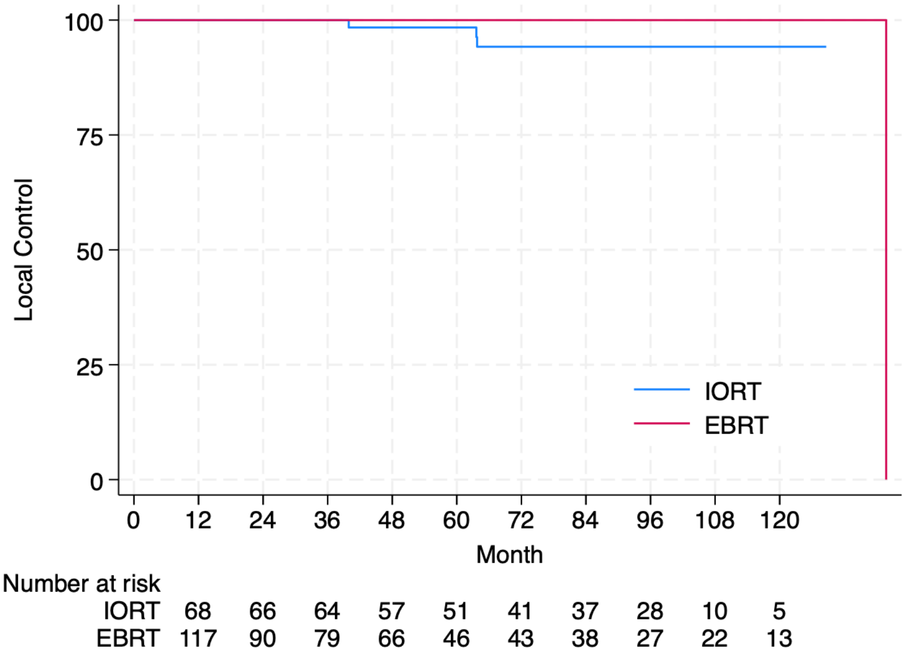

Supplementary Figure 2 Kaplan–Meier curve for local control among recommended and conditionally recommended patients per the 2023 ASTRO guideline on partial breast irradiation (*N* = 193; log-rank *P* = 0.08). Patients classified as conditionally not recommended per the 2023 American Society for Radiation Oncology guideline were excluded. The 5-year local control rates were 98.39% for the IORT group and 100% (no event) for the EBRT group. The number at risk at each time point is shown below the x-axis.

Abbreviations: ASTRO, American Society for Radiation Oncology; EBRT, external beam radiation therapy; IORT, intraoperative radiation therapy; LC, local control

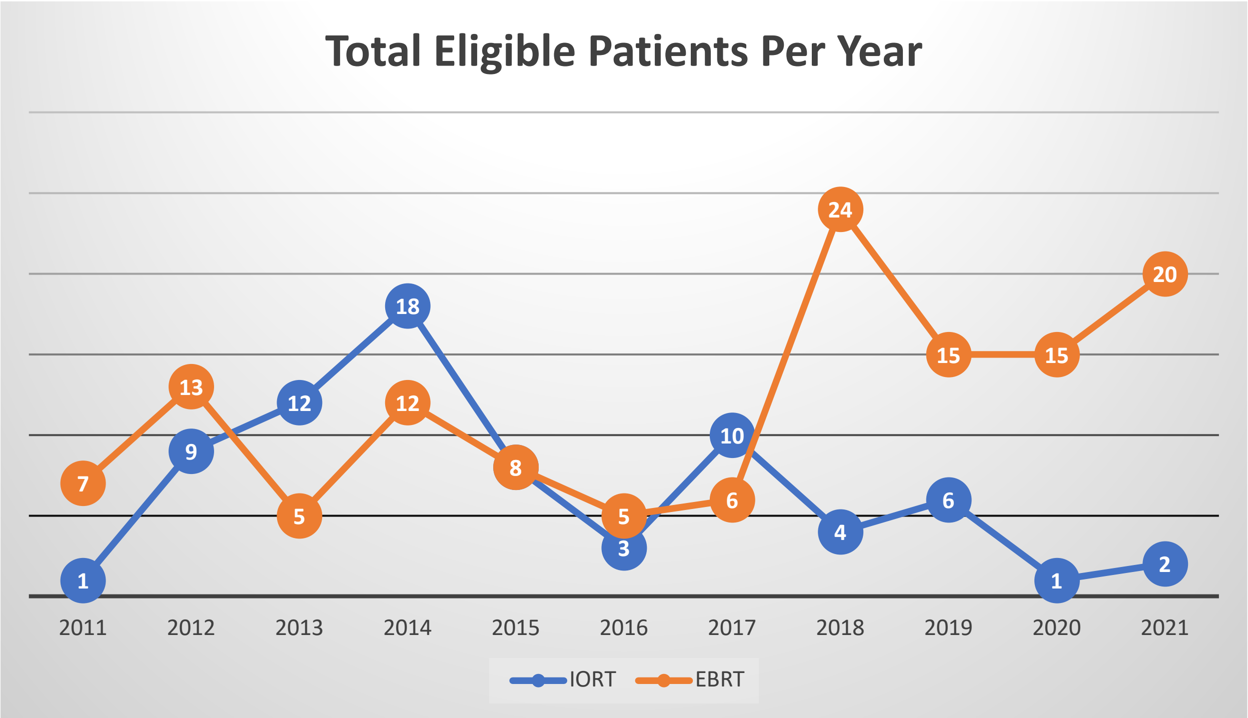

Supplementary Figure 3 Total eligible patients per year by radiation technique (*N* = 204).

Abbreviations: EBRT, external beam radiation therapy; IORT, intraoperative radiation therapy
